# Genotoxic Effects of Chromium(III) and Cobalt(II) and Their Mixtures on the Selected Cell Lines

**DOI:** 10.3390/ijms26115056

**Published:** 2025-05-24

**Authors:** Katarzyna Czarnek, Małgorzata Tatarczak-Michalewska, Eliza Blicharska, Andrzej K. Siwicki, Ryszard Maciejewski

**Affiliations:** 1 Institute of Medical Science, Faculty of Medical, The John Paul II Catholic University of Lublin, Konstantynów 1 H St., 20-708 Lublin, Poland; 2 Department of Pathobiochemistry and Interdisciplinary Applications of Ion Chromatography, Medical University of Lublin, 1 Chodźki St., 20-093 Lublin, Poland; malgorzatatatarczakmichalewska@umlub.pl (M.T.-M.); eliza.blicharska@umlub.pl (E.B.); 3 National Inland Fisheries Institute in Olsztyn, 10-917 Olsztyn, Poland; aksiw@infish.com.pl; 4 Institute of Health Science, Faculty of Medical, The John Paul II Catholic University of Lublin, Konstantynów 1 H St., 20-708 Lublin, Poland

**Keywords:** genome stability, cancer risk, genotoxic effects, chromium(III), cobalt(II), BALB/3T3, HepG2, the comet assay, the micronucleus assay

## Abstract

Cr(III) and Co(II) can be potentially toxic to cells and induce a number of morphological and biochemical changes. These metals are widely used in many industries and can cause environmental pollution. They are the components of dietary supplements, vitamin and mineral products, and energy drinks. Moreover, these metals are used in dentistry and orthopedics as components of implants. Data about the mechanism of genotoxic effects of Cr(III) and Co(II) are still incomplete. The aim of this study was to analyze the genotoxic effects of chromium(III) and cobalt(II) and their mixtures on two cell lines: mouse embryo fibroblast cell line BALB/3T3 and human hepatocellular carcinoma cell line G2 (HepG2). The BALB/3T3 and HepG2 cell lines were exposed to chromium chloride and cobalt chloride at concentrations ranging from 100 to 1400 µM. The genotoxicity assays used were the comet and micronucleus assays. On the basis of the results obtained from the first stage of the research, the concentrations of elements were selected in order to determine the interactions between them. The tested cell lines were treated with mixtures of the following compounds: chromium chloride at the concentration of 200 μM and cobalt chloride at the concentration of 1000 μM or chromium chloride at the concentration of 1000 μM and cobalt chloride at the concentration of 200 μM in the genotoxicity assays. This study shows that both cobalt(II) and chromium(III) cause genotoxic effects in the BALB/3T3 and HepG2 cell lines. A statistically significant increase in the percentage of comets was observed with increasing concentrations of Co(II) and Cr(III) compared to the control. A statistically significant induction of chromosomal aberrations was also observed in the micronucleus test. Moreover, chromium(III) at a concentration of 200 µM had a protective effect against the toxic concentration of cobalt(II) at a concentration of 1000 µM. The toxic effect of cobalt chloride and chromium chloride was confirmed in this study. Further research is needed on the genotoxic effects of cobalt(II) and chromium(III), especially due to the growing popularity of dietary supplements containing compounds of these metals and doubts as to the safety of their use.

## 1. Introduction

Humans are exposed to chromium and cobalt from the environment, from industry, and from the wear products of orthopedic and dental implants made from alloys of these metals. Cobalt(II) is a biologically essential trace element (TE) for humans, but only in a small amount below 1 µg/g of wet tissue [1]. TEs are essential for normal physiological function; they play a role in the prevention of nutritional deficiencies, the regulation of gene expression, the functioning of the immune and antioxidant defense systems, and the prevention of chronic diseases [2]. Cobalt is considered to be an essential trace element as it is a critical component of vitamin B12. The average daily dietary intake in adults is from 0.13 to 0.48 μg/kg body weight, and the physiologic blood concentrations are below 6 μg/L. As the active center of vitamin B12, cobalt participates in the metabolism of ribonucleic acid and hematopoietic substances. In addition, cobalt is involved in metabolism modulating transcriptional activator hypoxia-inducible factor-1 (HIF-1), which stimulates erythropoietin production [2,3,4]. Moreover, Co(II) is very important for forming amino acids and some proteins to create myelin sheath in nerve cells [5].

Chromium (Cr) may exist in different oxidation states, with Cr(III) and Cr(VI) being relatively stable and largely predominant. Cr(VI) is seldom found in nature and it mainly derives from industrial and anthropogenic activities. Hexavalent chromium is used in tanneries or in the metalworking industry, welding of stainless steel, and the production of chromates and chromium pigments. It is a known carcinogen absorbed through inhalation and associated with lung, nasal, and sinus cancers [6,7,8]. Cr(VI) is classified by the International Agency for Research on Cancer (IARC) as a human carcinogen (class I) [9].

For many years, Cr(III) was considered an essential trace element necessary for the normal metabolism of carbohydrates, fats, and proteins. However, recent research has challenged this view, leading to some re-evaluation of its status as an essential nutrient [10]. In 2014, the European Food Safety Authority (EFSA) determined that “no evidence of beneficial effects associated with chromium intake in healthy subjects” exists. Therefore, the EFSA issued a recommendation that no reference intake standards should be established for this element [10,11]. Currently, chromium can only be considered as pharmacologically active, not as an essential element.

Despite the lack of clear evidence of the beneficial effects associated with chromium intake, the position of Cr in the dietary supplement market is very strong. It is still one of the most popular trace elements consumed in the form of various products, especially by people who are overweight, obese, or have dyslipidemia or type-2 diabetes issues. Supplements containing Cr(III) are mainly used to reduce body weight and appetite for sweets [12]. Numerous studies point to major concerns about the effectiveness of Cr supplementation in the treatment of obesity and its related ailments; however, the authors do not rule out small positive health effects [13].

Chromium ions can be absorbed by the body orally, percutaneously or by inhalation, but the efficiency of absorption depends mainly on the degree of oxidation of these compounds—chromium(VI) is much more efficiently absorbed than its trivalent form [14]. Under physiological conditions, it is not possible to oxidize the trivalent form of chromium to hexavalent, whereas the inverse process of reducing Cr(VI) to Cr(III) normally occurs in organism cells. As a result of this process, many chromium intermediates can be generated, such as chromium(V) and chromium(IV), as well as reactive oxygen species (ROS) which damage cell biomolecules [14,15].

Excess Cr(III) and Co(II) can cause toxic effects in the human body [16,17,18,19,20,21,22]. Many studies report that cobalt and its compounds are carcinogenic for humans and are included in group 2 by the International Agency for Research on Cancer [23,24,25]. Chromium(III) and cobalt(II) are commonly widespread in water, air, and soil. In addition, these metals are widely used in many industries and can cause environmental pollution. They are the components of dietary supplements, vitamin and mineral products, and energy drinks [12,26,27,28,29,30,31,32]. Moreover, these metals are used in dentistry and orthopedics as components of orthopedic and trauma implants, crowns and bridges, as well as “stifts” for mounting porcelain crowns. According to literature data, the biomaterials used in implants can corrode in the environment of tissue fluids and the metal ions that are released in this way can be stored for a long time and can become toxic to organisms [33,34,35].

The environment of the oral cavity is very favorable to corrosion, with many factors including at least natural agents (saliva, water, and air), food contents, sugary drinks, dental plaque, microorganisms, very frequent pH, and temperature variations. Corrosion is the result of the oxidation of the metal parts. This phenomenon is multifactorial, and many types of corrosion can occur including pitting, fretting, and galvanic corrosion. When the corrosion process starts, Co and Cr ion metals are then released into the oral cavity [35]. The high mass of materials used to manufacture implants, especially orthopedic ones, bring a high risk of exposure to the metals. The products released into the peri-implant tissue are cations and metal nanoparticles, which are then distributed systemically [36,37,38]. Concentrations of metal ions in blood samples of patients with acute poisoning can reach up to 400–640 μg/L for cobalt and 50–80 μg/L for chromium, but concentrations of 2–7 μg/L are already considered to indicate excessive wear of the implant [39,40]. Peri-implant concentrations of metal ions can exceed these values, up to 397.8 mg/L Co, and show a shift to a highly increased Cr concentration. This is due to the generation of stable CrPO_3_ salts that are retained in the peri-implant tissue [36,37,40].

Cobalt(II) and chromium(III) can enter the body via the skin, through the respiratory and digestive systems, and as a component of biomaterials. No matter which way the metals enter the organism, they are always bound to proteins or blood cells in the bloodstream. Cobalt(II) is bound to red blood cells, while chromium(III) is bound to erythrocytes, polymorphonuclear and mononuclear leucocytes, and thrombocytes. Next, the metals are transported to organs, tissues, and cells where they are involved in their metabolism. These metals in high concentrations can be potentially toxic to cells and induce a number of morphological and biochemical changes leading to cell death by apoptosis. Accumulation of cobalt(II) in the cell can lead to interactions between the metal and DNA and nuclear proteins. According to literature data, exposure to cobalt or its compounds causes genotoxic effects on many cells. Some studies report that cobalt induces single and double-strand breaks, chromosome aberrations, and sister chromatid exchanges [41,42]. Additionally, cobalt causes small apoptotic bodies associated with the DNA fragmentation and its metal disrupts the normal course of the cell cycle [43,44,45,46,47]. Moreover, cobalt can cause an increase in protein kinase mitogen activated protein (MAP) and its phosphorylation and it elevates p53 protein levels, consequently affecting hypoxia-inducible factor-1 (HIF-1) activation, which leads to apoptosis. Furthermore, cobalt(II) destabilizes the proper functioning of enzymes belonging to the antioxidant system [41,48].

Chromium(III) is also a toxic metal that can bind to DNA in vitro forming Cr–DNA complexes and DNA–DNA cross-links [49]. Moreover, chromium causes DNA fragmentation, formation of micronuclei, induction of chromosome aberrations, and exchange of sister chromatids [50]. Additionally, chromium can bind to DNA and interfere with the replication process or block it completely [49].

The literature data clearly indicate that high concentrations of chromium(III) and cobalt(II), as well as their compounds, have a destructive effect on cell metabolism. Both metals are able to generate reactive oxygen species (ROS) that are very destructive in the DNA and other biomolecules. Moreover, they have a destructive effect on the antioxidant system, which is unable to remove harmful forms of oxygen under conditions of imbalance in the cell [51,52,53].

In the present study, the genotoxic effects of cobalt and chromium and their combinations were investigated in BALB/3T3 and HepG2 cell lines. Two cell lines, BALB/3T3 and HepG2, were exposed to chromium chloride and cobalt chloride at concentrations ranging from 100 to 1400 µM. The genotoxicity assays used were the comet and micronucleus assays. On the basis of the results obtained from the first stage of the research, the concentrations of elements were selected in order to determine the interactions between them. The tested cell lines were treated with mixtures of the following compounds: chromium chloride at the concentration of 200 μM and cobalt chloride at the concentration of 1000 μM or chromium chloride at the concentration of 1000 μM and cobalt chloride at the concentration of 200 μM in the genotoxicity assays.

## 2. Results

This study shows that both cobalt and chromium exhibit genotoxic properties by causing DNA and chromosome breaks and damage of the division spindle.

### 2.1. The Comet Assay

Both tested compounds caused DNA damage in the BALB/3T3 and HepG2 cell lines compared to the control.

In all tested lines, a statistically significant increase in the percentage of comets with an increasing concentration of the tested compound compared to the control was observed at a concentration of 400 µM.

**Figure 1:**
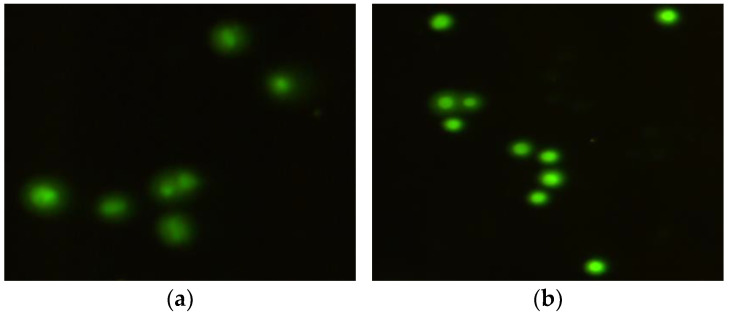
Control cells of the human hepatocellular carcinoma cell line G2 (HepG2) (**a**) and the mouse embryonic fibroblast (BALB/3T3) (**b**) cell lines; 200× magnification.

**Figure 2:**
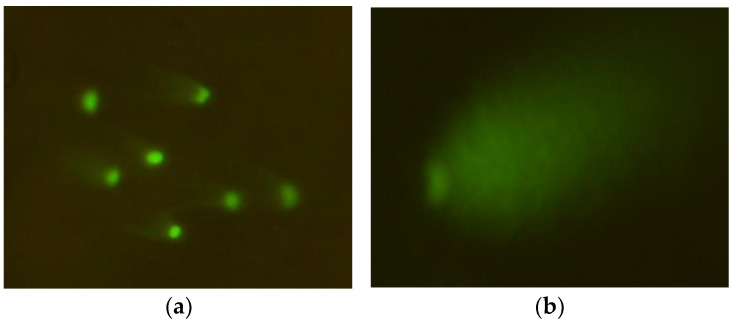
Typical images of comets observed in the mouse embryonic fibroblast cell line (BALB/3T3), created after exposure to 1000 µM CrCl_3_ × 6H_2_O; (**a**) 200× magnification; (**b**) 400× magnification.

An increase in the number of comets was observed for all lines treated with chromium chloride compared to the control (Table 1 and Table 2; Figure 1 and Figure 2).

**Figure 3:**
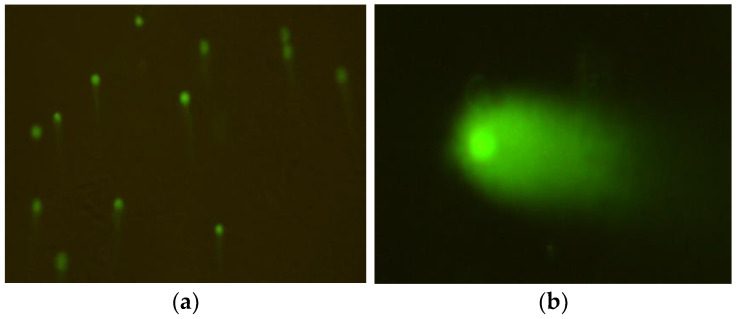
Typical images of comets observed in the human hepatocellular carcinoma cell line G2, created after exposure to 1000 µM CoCl_2_ × 6H_2_O; (**a**) 200× magnification; (**b**) 400× magnification.

**Figure 4:**
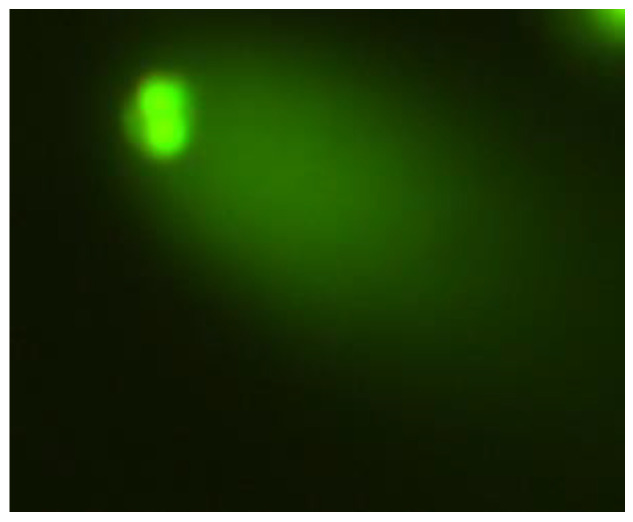
Typical image of comet observed in the human hepatocellular carcinoma cell line G2, created after exposure to 1200 µM CoCl_2_ × 6H_2_O; 400× magnification.

After incubation of mouse fibroblasts with cobalt chloride at the lowest concentrations, i.e., 100 and 200 µM, no comets were observed (Table 1). In the BALB/3T3 cell line, the DNA damage was visible as a comet tail at concentrations of 400 and 600 µM, respectively. In the concentration range from 800 to 1400 µM, a statistically significant increase in the percentage of comets was observed. After incubation of the HepG2 cell line with cobalt chloride at concentrations ranging from 100 to 400 µM, no comets were recorded. Comets formed as a result of DNA damage were observed at concentrations of 600 and 800 µM, while in the concentration range from 1000 to 1400 µM (Figure 1, Figure 3 and Figure 4), a significant increase in the percentage of comets was observed (Table 2).

Table 1 shows the results obtained in the comet assay after treatment of the BALB/3T3 cell line with cobalt chloride or chromium chloride and their mixtures.

Table 2 shows the results obtained in the comet assay after treatment of the HepG2 cell line with cobalt chloride or chromium chloride and their mixtures.

In the comet test, during simultaneous incubation of BALB/3T3 and HepG2 cell lines with 200 µM chromium chloride and 1000 µM cobalt chloride, a statistically significant decrease in the percentage of comets was observed compared to cells incubated only with 1000 µM cobalt chloride, and a small statistically significant increase in the percentage of comets was observed compared to cells incubated only with 200 µM chromium chloride. In the case of simultaneous incubation of both tested lines with 1000 µM chromium chloride and 200 µM cobalt chloride, an increase in the percentage of comets in the cells was observed compared to the percentage of comets observed in the cells incubated only with 200 µM cobalt chloride, whereas in comparison to the cells incubated only with 1000 µM chromium chloride, no significant differences were observed in the percentage of comets formed in the cells (Table 1 and Table 2).

### 2.2. The Micronucleus Assay

**Figure 5:**
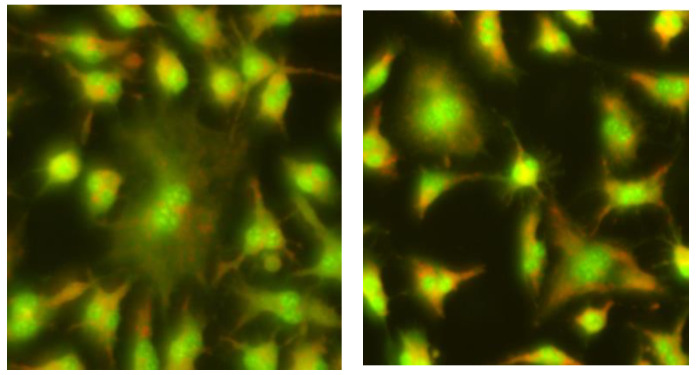
Typical images of mouse embryonic fibroblast (BALB/3T3) cells stained with acridine orange, observed under a fluorescence microscope, formed after exposure to 1000 µM CrCl_3_ × 6H_2_O; 200× magnification.

**Figure 6:**
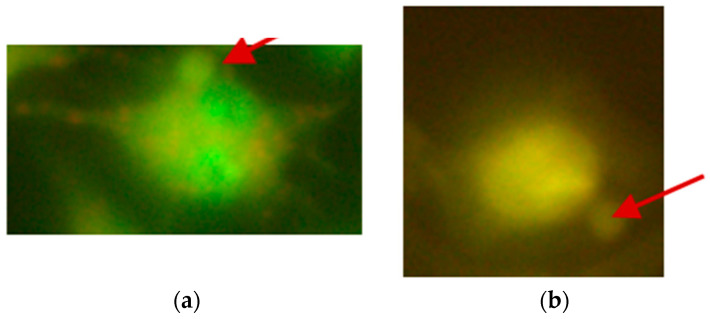
Micronuclei, observed in mouse embryonic fibroblast (BALB/3T3) (**a**) and human hepatocellular carcinoma cell line G2 (**b**) cell lines, formed after exposure to CrCl_3_ × 6H_2_O at a concentration of 1000 µM; 200× magnification.

In the micronucleus assay, during the incubation of the BALB/3T3 cell line with chromium chloride at the lowest concentration of 100 µM, no micronuclei were observed. An increase in the number of micronucleated cells was noted at a concentration of 200 µM, and a statistically significant increase in their number was observed with an increase in the concentration of the tested compound in the concentration range from 400 to 1000 µM compared to control cells of BALB/3T3 tested lines (Table 3; Figure 5 and Figure 6a).

In the HepG2 cell line, a slight increase in the number of micronucleated cells was observed at a concentration of 100 µM CrCl_3_ × 6H_2_O, and a statistically significant increase was observed in the concentration range from 200 to 1000 µM (Table 4; Figure 6b).

**Figure 7:**
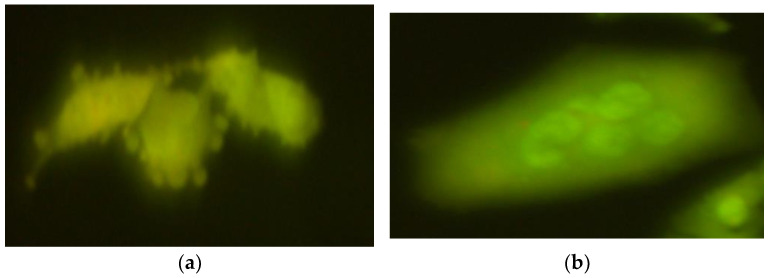
Typical images of mouse embryonic fibroblast (BALB/3T3) cells stained with acridine orange, observed under a fluorescence microscope, formed after exposure to 1200 µM CrCl_3_ × 6H_2_O; (**a**) 200× magnification; (**b**) 400× magnification.

**Figure 8:**
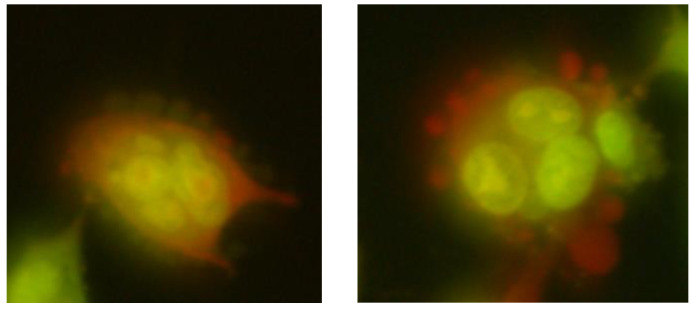
Typical images of human hepatocellular carcinoma cell line G2 stained with acridine orange, observed under a fluorescence microscope, formed after exposure to 1200 µM CrCl_3_ × 6H_2_O; 400× magnification.

In cells exposed to higher concentrations, i.e., 1200 and 1400 µM of chromium chloride, in both tested cell lines, multinucleated, giant cells with vacuolated cytoplasm and an altered membrane in the form of vesicles on its surface were observed. These changes indicate apoptosis (Table 3 and Table 4; Figure 7 and Figure 8).

**Figure 9:**
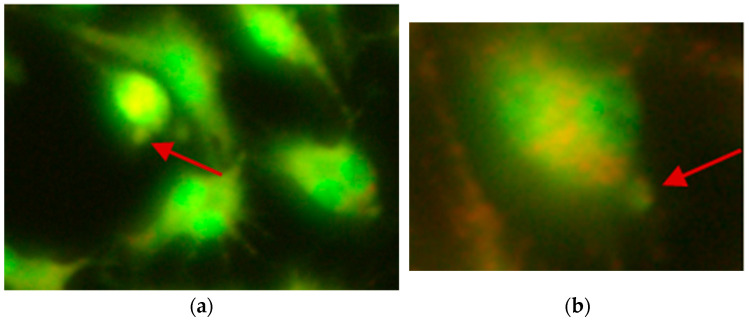
Micronuclei, observed in mouse embryonic fibroblast BALB/3T3 (**a**) and human hepatocellular carcinoma cell line G2 (**b**) cell lines, formed after exposure to CoCl_2_ × 6H_2_O at a concentration of 1000 µM; 200× magnification.

**Figure 10:**
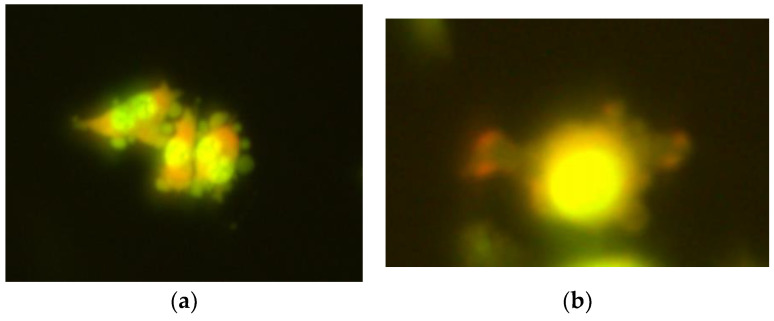
Typical images of mouse embryonic fibroblast BALB/3T3 cells stained with acridine orange, observed under a fluorescence microscope, formed after exposure to 1200 µM CoCl_2_ × 6H_2_O; (**a**) 200× magnification; (**b**) 400× magnification.

After the BALB/3T3 and HepG2 cell lines were exposed to cobalt chloride, a slight increase in the number of cells with micronuclei was observed at lower concentrations, with 1000 cells not counted in the fibroblast line at 400 µM. In the fibroblast line, multinucleated, giant cells with vacuolated cytoplasm and an altered membrane in the form of vesicles on its surface were observed at concentrations from 600 to 1400 µM, which indicates apoptosis. In the HepG2 line, such observations were made at concentrations from 400 to 1400 µM (Table 3 and Table 4; Figure 9 and Figure 10).

Simultaneous incubation of cells from both tested lines with 200 µM chromium chloride and 1000 µM cobalt chloride resulted in an increase in the number of cells with micronuclei compared to cells incubated with 200 µM chromium chloride, and an increase in the number of normal cells compared to cells incubated only with 1000 µM cobalt chloride. In the case of simultaneous incubation of cells from the tested lines with 1000 µM chromium chloride and 200 µM cobalt chloride, a statistically significant increase in the number of cells with micronuclei was noted compared to cells incubated only with 1000 µM chromium chloride or 200 µM cobalt chloride (Table 3 and Table 4).

## 3. Discussion

The toxic effects of cobalt(II) and chromium(III) on cells result in various morphological, biochemical, and functional alterations, including the degradation of proteins, lipids, and deoxyribonucleic acid, as well as disruptions to cellular metabolism, which can ultimately lead to cell death through apoptosis [48,54,55]. This study shows that both cobalt(II) and chromium(III) cause genotoxic effects in the BALB/3T3 and HepG2 cell lines. In this work, two cell lines, BALB/3T3 and HepG2, were exposed to chromium chloride and cobalt chloride at concentrations ranging from 100 to 1400 µM. The BALB/3T3 line is a line of mouse fibroblasts that is recommended by the European Union Reference Laboratory for Alternatives to Animal Testing (EURL ECVAM laboratory) for testing morphological and biochemical changes in cells caused by microelements. However, HepG2 is a human cancer line (hepatocellular carcinoma), recommended in the genotoxicity assay for micronucleus formation. This line is used to demonstrate the genotoxic activity of the tested substances. Moreover, the use of these lines was intended to indicate the differences between cancerous and normal cells.

In this study, the toxic effect of cobalt chloride and chromium chloride was confirmed using two genotoxicity assays: comet and micronucleus. In the comet assay, after incubation of the BALB/3T3 cell line at the lowest concentrations of cobalt chloride, no comets were observed. A slight increase in the comet percentage was detected at the concentrations of 400 µM and 600 µM, with a more pronounced increase at concentrations ranging from 800 µM to 1400 µM. For cancer cell lines, DNA damage, indicated by comet tails, was observed at 800 µM, and a significant increase in comet frequency was noted at concentrations between 1000 µM and 1400 µM. The results obtained in this study indicate that cobalt chloride causes the formation of DNA breaks and affects the formation of apurinic/apyrimidinic AP sites. The toxic effect of cobalt chloride was confirmed in studies conducted on the mouse astrocytes C57BL/6, human large cell lung carcinoma cell line H460, human adult keratinocyte cell line HaCaT, human lung fibroblasts WTHBF-6, and mouse macrophages J774. Exposure to cobalt(II) ions led to the induction of double-strand DNA breaks and the formation of small apoptotic bodies resulting from DNA fragmentation. Additionally, signs of late-stage apoptosis were observed, characterized by alterations in the cell membrane and the presence of apoptotic bodies, along with necrosis, which was marked by extensive damage to the cell membrane [46,49,56,57,58,59]. Moreover, literature data report that exposure to cobalt(II) compounds causes the formation of DNA breaks, the formation of cross-links between proteins and DNA, the exchange of sister chromatids, and the formation of micronuclei in mammalian cells [46,60].

In the present study, the genotoxicity effect of cobalt was confirmed in the micronucleus assay [61]. Additionally, giant multinucleated cells with vacuolated cytoplasm and an altered cell membrane in the form of vesicles on its surface were observed in the BALB/3T3 and HepG2 cell lines. These changes are the results of chromosomal breaks and spindle damage. The same observations were confirmed by other studies, which reported that MG-63 osteoblasts, when treated with cobalt(II) ions, have significantly increased in size, and the large spaces visible in the microscopic image indicate a significant decrease in cell number [45].

Similar observations were obtained in a clastogenic test, in which WTHBF-6 fibroblasts were exposed to cobalt chloride at concentrations of 100, 175, and 250 µM. Chromatid damage, numerous aberrations, and cell cycle arrest have been reported [56]. Moreover, cobalt(II) induces the formation of double breaks in DNA and mutations in genes [46,56,57,58].

This metal can induce oxidation and nitrification processes of cellular proteins which lead to apoptosis [62]. Additionally, cobalt(II) can inhibit the antioxidative activity of enzymes especially the superoxide dismutase, catalase, and glutathione reductase [41,63,64,65]. These observations were made in other studies in which the osteoblast line was treated with cobalt(II) ions. Moreover, we noticed some changes in the superoxide dismutase (SOD) enzyme activity depending on the time of cobalt exposure. Furthermore, the 11% decrease in the enzyme was observed after 24 hrs and 36% after 72 h. Regarding the heme oxygenase-1 (HO-1) enzyme, it reached the maximum stimulation of its activity during the first 24 h (six-fold higher compared to the control), and a gradual decrease in its activity was observed over this time. A slight increase in the glutathione peroxidase (GPx) activity was stimulated by cobalt [66].

Since cobalt can generate reactive oxygen species (ROS), its genotoxicity effects are obviously very destructive to DNA. Additionally, cobalt(II) can lead to DNA fragmentation and activate the caspase system when reacting with hydrogen peroxide in Fenton reaction. Then, the resulting free radicals can be destructive in cells. The degree of the free radicals formation is dependent on the condition of the defense system [41,63,64].

The enzyme activity is decreased due to a reduction in the SOD enzyme activity, which is associated with the binding of cobalt(II) to the zinc and copper sites in SOD, thereby reducing the enzyme activity. Similarly, cobalt(II) can bind to sulfhydryl groups of proteins and enzymes and interfere with the proper metabolism of glutathione—an essential antioxidant that plays a key role in metabolism and excretion of xenobiotics [67,68]. The DNA repair mechanisms can be affected by cobalt(II) through interactions with magnesium and zinc ions in enzymes. Inhibition of this process contributes to mutation formation [46,69]. Cobalt(II) can cause cell destruction, which leads to apoptosis [70]. One of the most important mechanisms, which are responsible for mutagenesis and carcinogenesis processes induced by cobalt(II) ions, is the inhibition of DNA repair processes. This element probably disrupts the proper functioning of the zinc finger domain of the xeroderma pigmentosum group A (XPA) protein, which is active in the process of nucleotide excision during the repair of mutated DNA. Disruption of the activity of this protein results in the inhibition of repair processes and duplication of mutated DNA [71]. Replacing zinc with cobalt(II) in the XPA protein promotes the formation of ROS near DNA, which affects its destruction [64].

According to Baldwin et al., the genotoxic effects induced by cobalt(II) may also be related to the inhibition of topoisomerase II α activity enzyme in animal cells. Cobalt(II) interferes with the proper functioning of topoisomerase II α by forming complexes with this enzyme [46]. Additionally, cobalt(II) competes with zinc ions affecting the efficiency of the mechanism of p53 protein binding to DNA, which is dependent on the presence of these ions [72,73]. Our studies also confirmed the genotoxic effect of chromium(III) chloride. In the comet assay, a statistically significant increase in the number of comets was observed in the BALB/3T3 and HepG2 cell lines with increasing chromium(III) salt concentrations. Literature data clearly indicate that chromium(III) is a genotoxic metal [43,44]. In this study, genotoxicity tests were conducted to confirm the destructive effect of the element on DNA. According to some literature data, chromium(III) can bind to DNA, and then adducts and cross-links between DNA strands can be formed [74]. This mechanism results from the reaction of Cr^3+^ ions with negatively charged phosphate groups or directly with guanine [75]. In the comet test detecting DNA breaks, a statistically significant increase in the number of comets was noted with increasing chromium chloride concentrations. These results are consistent with the studies by other authors, in which the BJ cell line was exposed to chromium chloride and the development of chromosomal aberrations in a dose-dependent manner was observed [53]. Additionally, it is confirmed by other studies that high concentrations of chromium(III) and its compounds, i.e., chromium picolinate and nicotinate, are capable of inducing chromosomal aberrations, causing DNA fragmentation in macrophages [43]. Chromium chloride can be destructive to DNA by forming breaks in double-stranded and single-stranded DNA and AP sites that lack purines and pyrimidines. If DNA damage is not removed at cell cycle checkpoints, the resulting mutations are replicated. Studies on the effect of chromium chloride on the cell cycle of BALB/3T3 and HepG2 lines are available in the literature. An analysis of the cycle checkpoints indicated that chromium chloride caused a decrease in the number of cells in the G0/Gl phase with increasing element concentration. In the G2/M phase, an increase in the number of cells that are unable to enter the mitotic division was observed [76]. Other authors indicate that chromium(III) can induce substitution, transversion, deletion, and insertion of mutations [73]. In the next test, micronucleus formation which allows for the detection of chromosome breaks and spindle damage, a statistically significant increase in the number of cells with micronuclei was noted for all tested lines. These results also confirm the previous studies in which chromium(III) complexes induced micronucleus formation in Chinese hamster ovary lung V79 cells and mouse collagenase resistant MCR fibroblasts [43,77]. In the results of the research presented in this paper, for all lines in the micronucleus test above a concentration of 1000 µM, giant multinucleated cells with vacuolated cytoplasm and an altered membrane in the form of vesicles on its surface were observed. These are cells in the subG1 phase that do not undergo normal divisions but replicate nuclei without dividing the cytoplasm. These results were confirmed by previous studies in which the phases of the cell cycle were analyzed and an increase in cells in the subG1 phase was noted after exposure to increasing concentrations of chromium chloride [76]. The microscopic observation carried out in this study allowed for the observation of cells with signs of apoptosis, which indicates the destructive effect of chromium chloride on the cells of all the lines studied [61]. These observations confirm previous studies in which keratinocytes exposed to chromium chloride or chromium picolinate showed features characteristic of apoptotic cells, i.e., chromatin condensation and the presence of numerous vesicles on the surface of the cell membrane [78,79]. As mentioned above, chromium(III) compounds can generate some reactive oxygen species, which in turn, can be destructive to the mitochondria of cells that may lead to apoptosis. These genotoxic effects of chromium(III) were observed in Chinese hamster AA8 cells [80]. Another effect of the destructive role of chromium(III) compounds is lipid peroxidation in liver and kidney cells. One of the products of its action is the formation of malondialdehyde (MDA)—a marker of oxidative stress in DNA-8-hydroxy-deoxy-guanosine [81]. It is formed as a result of the oxidation of guanine at the C8 position in the DNA strand by the hydroxyl radical. The formation of such a compound leads to the replacement of guanine with thymine during replication, and consequently to numerous mutations [81]. This element can bind to DNA and disrupt the replication process (in lower concentrations) or block it completely. The activity of SOD and glutathione peroxidase GSH-Px enzymes gradually decreases under the influence of an increase in reactive oxygen species, which leads to irreversible destructive changes, i.e., lipid peroxidation and the formation of malondialdehyde MDA [82]. Studies conducted by Terpiłowska and Siwicki (2019) indicate an increase in the concentration of free radicals and malondialdehyde above the concentration of 400 µM chromium chloride and an increase in the activity of superoxide dismutase and catalase at concentrations of 100 and 200 µM, which decreased above 400 µM. In the case of glutathione peroxidase, a decrease in enzyme activity was noted with an increase in the concentration of the element in the case of the BALB/3T3 and HepG2 lines [83]. Based on the results from the literature data, chromium(III) has a destructive effect on the antioxidant system, which is unable to remove harmful forms of oxygen in conditions of disturbed equilibrium in the cell. Chromium(III) causes the destructive effects while destructing the active center of the enzyme. The activity of superoxide dismutase and glutathione peroxidase enzymes gradually decreases under the influence of ROS, which leads to irreversible destructive changes, i.e., lipid peroxidation and the formation of malondialdehyde [79,81,84,85]. In some other studies, in which the osteoblast line was exposed to cobalt(II) ions for 24 to 72 hrs, changes in the activity of antioxidant enzymes were noted, followed by an increase in catalase (CAT) activity, which reached a value of 1.7 times higher and GPx 1.9 times higher compared to the control system after 72 h of incubation of cells with metal. In the case of the enzyme heme oxygenase, a maximum stimulation of its activity was noted within the first 24 h (two times higher compared to the control), followed by a gradual decrease in activity over time [50].

The impact of elements, i.e., their interactions, can be of a different nature. In the case of a decrease in the effects of the elements tested, antagonism is observed, and in the case of their intensification, synergism. During simultaneous incubation with chromium chloride at a concentration of 200 µM and cobalt chloride at a concentration of 1000 µM of cells of all tested lines, antagonism was observed—chromium(III) at a concentration of 200 µM performed protective functions against the toxic concentration of cobalt(II) at a concentration of 1000 µM. Observations were made in genotoxicity tests, wherein a decrease in the percentage of comets and the number of mutations was noted. In the case of the micronucleus formation test, an increase in the number of normal cells was noted, which is consistent with the protective effect of chromium(III) in this interaction at a concentration of 200 µM. The results obtained in this work confirm the studies conducted by other authors. In BALB/3T3 and HepG2 cell lines exposed to chromium chloride at a concentration of 100 and 200 µM, an increase in the activity of antioxidant enzymes, catalase and peroxidase, was observed [83]. These studies showed that the activity of catalase and superoxide dismutase in cells exposed to chromium chloride above a concentration of 400 µM decreased. Our results are similar to other studies conducted by Chen et al., in which enzymes of the antioxidant system (in a non-cellular system) were exposed to chromium(III) in the range from 0 to 5.0 × 10^−4^ mol L^−1^. At low concentrations, i.e., 2.0 × 10^−4^ mol L^−1^ of chromium chloride, an increase in catalase activity was observed, which decreased at higher concentrations. Chromium(III) causes the destructive effect while destructing the active center of the enzyme [72]. The possibility of stimulating catalase activity by low concentrations of chromium(III) results in its protective effect against the post-oxidative effect of cobalt(II) used in high concentrations. Still, no protective effect of chromium chloride against cobalt chloride was observed in the case of the interaction of chromium chloride at a concentration of 1000 µM and cobalt chloride at a concentration of 200 µM. Here, synergism between the tested elements was noticed. Moreover, an increase in the percentage of comets and an increase in the number of comets, but at the same time, a decrease in the number of normal cells were noted. The lack of protective effect of chromium chloride at a concentration of 1000 µM is associated with the generation of free oxygen radicals and a decrease in the efficiency of the antioxidant system [72]. In the case of the interaction of chromium(III) at a concentration of 1000 µM with cobalt chloride at a concentration of 200 µM, the former compound enhances the proapoptotic effect of cobalt(II).

## 4. Materials and Methods

### 4.1. Reagents

Chromium chloride (CrCl_3_ × 6H_2_O) and cobalt chloride (CoCl_2_ × 6H_2_O) were purchased from Across Organics (Geel, Belgium); Eagle’s Minimum Essentials Medium (EMEM) and fetal bovine serum (FBS) were obtained from American Type Culture Collection (ATCC); Dulbecco’s Modified Eagle Medium (DMEM) and calf bovine serum (CBS) were obtained from ATCC; antibiotic antimycotic solution (10,000 U/mL of penicillin, 10 mg/mL of streptomycin, 25 µg/mL of amphotericin B), acridine orange, and cytochalasin B were purchased from Sigma Chemical Co. (St. Louis, MO, USA). Oxi select 96-well comet assay kit was purchased from Cell Biolabs, Imc., San Diego, CA, USA.

The research was carried out on the mouse fibroblast BALB/3T3 cell lines obtained from American Type Culture Collection (CCL-163). The cells were grown as adherent monolayers in plastic tissue culture dishes in Dulbecco’s Modified Eagle Medium (DMEM), which was supplemented with 10% calf bovine serum (CBS) and antibiotic antimycotic solution (10,000 U/mL of penicillin, 10 mg/mL of streptomycin, 25 µg/mL of amphotericin B) at 37 °C and 5% CO_2_.

The research was carried out on HepG2 cell lines obtained from American Type Culture Collection (HB-8065). The cells were grown as adherent monolayers in plastic tissue culture dishes in Eagle’s Minimum Essentials Medium (EMEM), which was supplemented with 10% fetal bovine serum (FBS) and antibiotic antimycotic solution (10,000 U/mL of penicillin, 10 mg/mL of streptomycin, 25 µg/mL of amphotericin B) at 37 °C and 5% CO_2_.

Chromium chloride (CrCl_3_ × 6H_2_O) and cobalt chloride(CoCl_2_ × 6H_2_O) were dissolved in PBS at the concentration of 1 mM. A solution of chromium chloride or cobalt chloride at a concentration range from 100 to 1400 µM was prepared by dilution in culture medium in the case of BALB/3T3 DMEM supplemented with CBS and antibiotics and in the case of HepG2 EMEM supplemented with FBS and antibiotics.

In the genotoxicity assays, the BALB/3T3 and HepG2 cell lines were exposed to chromium chloride and cobalt chloride at concentrations ranging from 100 to 1400 µM.

### 4.2. Genotoxicity Assays

The methodology used was the same as in [61]. In this work, the comet and micronucleus assays were used. The cells were cultured in 96-well plates (2 × 10^5^ cells/mL) in 100 µL in complete growth medium (DMEM supplemented with 10% CBS and a mixture of antibiotics) or (EMEM supplemented with 10% FBS and a mixture of antibiotics). After 24 h of incubation of BALB/3T3 and HepG2, the culture fluid was exchanged into a new one in the control case or supplemented with chromium chloride or cobalt chloride at concentrations ranging from 100 to 1400 µM. In order to determine the interactions between the above-mentioned microelements, the cells were similarly plated and incubated for 24 h. Next, the culture fluid was exchanged for the fresh one and supplemented with mixtures of the following compounds: 200 µM chromium chloride and 1000 µM cobalt chloride (Cr200+Co1000) or 1000 µM chromium chloride and 200 µM cobalt chloride (Cr1000+Co200).

#### 4.2.1. The Comet Assay

The comet assay is a common technique for the measurement of DNA damage in individual cells. This assay detects breaks in DNA. Nucleic acid fragments subjected to electrophoresis move outside the nucleus forming a comet-like tail [86]. This assay was performed according to the original manufacturer’s instructions—Oxi Select 96-Well Comet Assay Kit, (Cell Biolabs, Inc., USA).

The prepared plates were subjected to microscopic observation using a fluorescein isothiocyanate (FITC) filter. One hundred cells were selected from each concentration, and then the cells with damaged DNA, the so-called ones with a comet tail, were photographed with the use of the image analysis program Leica Application Suite 4.4 using a Leica DM IL LED FLUO (Leica Microsystems, Wetzlar, Germany) inverted microscope. The experiment was performed in 6 independent replications.

#### 4.2.2. The Micronucleus Assay

This assay is used to detect chromosome breaks and division spindle defects. Micronuclei are formed from the acentric chromatids, chromosome fragments, or from whole chromosomes. These fragments after telephosis are not incorporated into the nuclei of progenitor cells and they form micronuclei in the cytoplasm [87]. The micronucleus test was performed in accordance with OECD Guideline 487 [88] and PN-EN ISO 10993-3:2014 standard.. After 24 h of incubation of the cells with the test compounds or their mixtures, cytochalasin B was added. Then, the culture fluid was decanted from the plates and the dye, acridine orange, was added at the final concentration of 100 µg/mL. Microscopic observations were then made using the appropriate FITC filter and photographic documentation was made using the image analysis software Leica Application Suite 4.4 using a Leica DM IL LED FLUO inverted microscope. The results were reported by analyzing the binucleated micronucleated cell frequency as a number of binucleated cells containing one or more micronuclei per 1000 binucleated cells. The experiment was performed in 6 independent replications.

### 4.3. Statistical Analysis

The results were analyzed using one-way analysis of variance (ANOVA) with Tukey’s multiple comparisons using Statistica version 4.0. In all cases, *p* < 0.05 was considered significant.

## 5. Conclusions

Cobalt and chromium in high concentrations can be potentially toxic to cells and induce a number of morphological and biochemical changes leading to cell death by apoptosis. Accumulation of cobalt and chromium in the cell can lead to interactions between the metal and DNA and nuclear proteins.

In this study, a statistically significant increase in the percentage of comets was observed with increasing concentrations of Co(II) and Cr(III) compared to the control. In the micronucleus assay, a statistically significant induction of chromosomal aberrations in the above-mentioned compounds was also observed.

During simultaneous incubation with chromium chloride at a concentration of 200 µM and cobalt chloride at a concentration of 1000 µM of tested cell lines, antagonism was observed—chromium(III) at a concentration of 200 µM performed protective functions against the toxic concentration of cobalt(II) at a concentration of 1000 µM. Observations were made in the genotoxicity tests, wherein a decrease in the percentage of comets and the number of mutations was noted. In the case of the micronucleus formation test, an increase in the number of normal cells was noted.

The toxic effect of cobalt chloride and chromium chloride was confirmed in this study. Further research is needed on the genotoxic effects of cobalt(II) and chromium (III), especially due to the growing popularity of dietary supplements containing compounds of these metals and doubts as to the safety of their use.

## Figures and Tables

**Table 1:** Percentage of DNA tail after incubation with chromium chloride or cobalt chloride and their mixtures in the mouse embryonic fibroblast cell line (BALB/3T3), detected with the comet assay.

Concentration [µM]		Mean [%] Tail DNA ± SD
CrCl_3_ × 6H_2_O		
0		0 ± 0.0
100		2 ± 0.0
200		3 ± 0.2
400		4 ± 0.3 *
600		5 ± 0.4 *
800		6 ± 0.6 *
1000		7 ± 0.6 *
1200		11 ± 1.0 *
1400		13 ± 1.0 *
CoCl_2_ × 6H_2_O		
0		0 ± 0.0
100		0 ± 0.0
200		0 ± 0.0
400		2 ± 0.2 *
600		4 ± 0.3 *
800		75 ± 6.5 *
1000		84 ± 7.4 *
1200		98 ± 8.5 *
1400		99 ± 8.1 *
CrCl_3_ × 6H_2_O and CoCl_2_ × 6H_2_O mixtures		
200 µM CrCl_3_ × 6H_2_O	1000 µM CoCl_2_ × 6H_2_O	10 ± 1.0 *^1,2^
1000 µM CrCl_3_ × 6H_2_O	200 µM CoCl_2_ × 6H_2_O	7 ± 0.6 *

* significant difference compared with control, *p* ≤ 0.05; ^1^ significant difference compared with chromium chloride at a concentration of 200 µM, *p* ≤ 0.05; ^2^ significant difference compared with cobalt chloride at a concentration of 1000 µM, *p* ≤ 0.05.

**Table 2:** Percentage of DNA tail after incubation with chromium chloride or cobalt chloride and their mixtures in the human hepatocellular carcinoma cell line G2 (HepG2), detected with the comet assay.

Concentration [µM]		Mean [%] Tail DNA ± SD
CrCl_3_ × 6H_2_O		
0		0 ± 0.0
100		2 ± 0.0
200		3 ± 0.0
400		3 ± 0.3 *
600		3 ± 0.3 *
800		3 ± 0.3 *
1000		4 ± 0.3 *
1200		6 ± 0.5 *
1400		9 ± 0.9 *
CoCl_2_ × 6H_2_O		
0		0 ± 0.0
100		0 ± 0.0
200		0 ± 0.0
400		0 ± 0.0
600		1 ± 0.1
800		10 ± 1.0 *
1000		97 ± 8.9 *
1200		98 ± 8.3 *
1400		100 ± 9.1 *
CrCl_3_ × 6H_2_O and CoCl_2_ × 6H_2_O mixtures		
200 µM CrCl_3_ × 6H_2_O	1000 µM CoCl_2_ × 6H_2_O	17 ± 1.0 *^1,2^
1000 µM CrCl_3_ × 6H_2_O	200 µM CoCl_2_ × 6H_2_O	5 ± 0.4 *

* significant difference compared with control, *p* ≤ 0.05; ^1^ significant difference compared with chromium chloride at a concentration of 200 µM, *p* ≤ 0.05; ^2^ significant difference compared with cobalt chloride at a concentration of 1000 µM, *p* ≤ 0.05.

**Table 3:** Frequency of micronucleated binucleated cells induced by chromium chloride or cobalt chloride and their mixtures in the mouse embryonic fibroblast cell line (BALB/3T3), detected with the micronucleus assay.

Concentration [µM]		BNMN‰
CrCl_3_ × 6H_2_O		
0		0 ± 0.0
100		0 ± 0.0
200		2 ± 0.0
400		6 ± 0.5 *
600		12 ± 1.0 *
800		13 ± 1.0 *
1000		22 ± 1.9 *
1200		apoptosis
1400		apoptosis
CoCl_2_ × 6H_2_O		
0		0 ± 0.0
100		1 ± 0.0
200		3 ± 0.2 *^a^
400		4 ± 0.4 *^a^
600		apoptosis
800		apoptosis
1000		apoptosis
1200		apoptosis
1400		apoptosis
CrCl_3_ × 6H_2_O and CoCl_2_ × 6H_2_O mixtures		
200 µM CrCl_3_ × 6H_2_O	1000 µM CoCl_2_ × 6H_2_O	20 ± 1.8 *^1,2^
1000 µM CrCl_3_ × 6H_2_O	200 µM CoCl_2_ × 6H_2_O	30 ± 0.2 *^3,4^

* significant difference compared with control *p* ≤ 0.05; ^a^ 1000 cells were not counted; ^1^ significant difference compared with chromium chloride at a concentration of 200 µM, *p* ≤ 0.05; ^2^ significant difference compared with cobalt chloride at a concentration of 1000 µM, *p* ≤ 0.05; ^3^ significant difference compared with chromium chloride at a concentration of 1000 µM, *p* ≤ 0.05; ^4^ significant difference compared with cobalt chloride at a concentration of 200 µM, *p* ≤ 0.05.

**Table 4:** Frequency of micronucleated binucleated cells induced by chromium chloride or cobalt chloride and their mixtures in the human hepatocellular carcinoma cell line G2 (HepG2), detected with the micronucleus assay.

Concentration [µM]		BNMN‰
CrCl_3_ × 6H_2_O		
0		0 ± 0.0
100		3 ± 0.0
200		4 ± 0.3 *
400		5 ± 0.4 *
600		10 ± 1.0 *
800		10 ± 1.0 *
1000		16 ± 0.9 *
1200		apoptosis
1400		apoptosis
CoCl_2_ × 6H_2_O		
0		0 ± 0.0
100		1 ± 0.0
200		2 ± 0.2 *
400		apoptosis
600		apoptosis
800		apoptosis
1000		apoptosis
1200		apoptosis
1400		apoptosis
CrCl_3_ × 6H_2_O and CoCl_2_ × 6H_2_O mixtures		
200 µM CrCl_3_ × 6H_2_O	1000 µM CoCl_2_ × 6H_2_O	30 ± 2.8 *^1,2^
1000 µM CrCl_3_ × 6H_2_O	200 µM CoCl_2_ × 6H_2_O	20 ± 2.0 *^3,4^

* significant difference compared with control *p* ≤ 0.05; ^1^ significant difference compared with chromium chloride at a concentration of 200 µM, *p* ≤ 0.05; ^2^ significant difference compared with cobalt chloride at a concentration of 1000 µM, *p* ≤ 0.05; ^3^ significant difference compared with chromium chloride at a concentration of 1000 µM, *p* ≤ 0.05; ^4^ significant difference compared with cobalt chloride at a concentration of 200 µM, *p* ≤ 0.05.

## Data Availability

Data are contained within the article.
